# Epigenetic silencing of NKD2, a major component of Wnt signaling, promotes breast cancer growth

**DOI:** 10.18632/oncotarget.4244

**Published:** 2015-06-19

**Authors:** Yan Dong, Baoping Cao, Meiying Zhang, Weidong Han, James G. Herman, François Fuks, Yali Zhao, Mingzhou Guo

**Affiliations:** ^1^ Department of Gastroenterology & Hepatology, Chinese PLA General Hospital, Beijing 100853, China; ^2^ Department of Molecular Biology, Institute of Basic Medicine, School of Life Sciences, Chinese PLA General Hospital, Beijing 100853, China; ^3^ Medical College of NanKai University, Tianjin 300071, China; ^4^ The Hillman Cancer Center, University of Pittsburgh Cancer Institute, Pittsburgh, PA 15213, U.S.A; ^5^ Laboratory of Cancer Epigenetics, Free University of Brussels (U.L.B.), 808 route de Lennik, Brussels 1070, Belgium; ^6^ Central Laboratory, The Affiliated Hainan Hospital of Chinese PLA General Hospital, Hai Tang Wan, Sanya 572013, China

**Keywords:** NKD2, DNA methylation, Wnt signaling, epigenetics, breast cancer

## Abstract

Naked cuticle homolog 2 (NKD2) has been reported to antagonize Wnt signaling in zebrafish, mouse and mammals. The aim of this study is to investigate the epigenetic changes and mechanisms of NKD2 in human breast cancer development. Six breast cancer cell lines (MCF-7, ZR75-1, MDA-MB-468, MDA-MB-231, T47D and BT474) and 68 cases of primary human breast cancer were studied using methylation specific PCR, immunohistochemistry, western blot, flow cytometry techniques and a xenograft mouse model. The expression of NKD1 and NKD2 was regulated by promoter region methylation in breast cancer cells. No NKD1 methylation was found in primary human breast cancer. NKD2 was methylated in 51.4% (35/68) of human primary breast cancer samples. NKD2 methylation was significantly associated with reduction of NKD2 expression, and tumor stage (*p* < 0.05). NKD2 suppressed breast cancer cell proliferation both *in vitro* and *in vivo*. NKD2 induced G1/S arrest and inhibited Wnt signaling in breast cancer cells. In conclusion, NKD2 is frequently methylated in human breast cancer, and the expression of NKD2 is regulated by promoter region methylation. NKD2 suppresses breast cancer proliferation by inhibiting Wnt signaling.

## INTRODUCTION

Breast cancer is the most common malignancy in women worldwide, and it is the second leading cause of cancer-related death among women [1]. The incidence of breast cancer is much lower in China than in Western countries [2]. The geographical variation indicates that environmental factors may play a significant role in the risk of breast cancer development. Epigenetic changes are strongly influenced by environmental factors. Aberrant epigenetic changes have been frequently reported in human breast cancer [3]. Wnt signaling has been reported to play important roles in mammary gland development and breast tumorigenesis [4]. Genetic mutations in *APC* and *CTNNB1* (β-catenin encoding gene) are major contributors to colorectal carcinogenesis. However, genetic mutations in these genes are not key contributors to breast cancer development [5, 6]. It has been demonstrated that only 6% of breast cancers harbor mutations in the *APC* gene, and no mutations in *CTNNB1* have been identified in breast cancer [7, 8]. Therefore, epigenetics may play an important role in breast cancer development.

The naked cuticle (NKD) family includes Drosophila naked cuticle and its two vertebrate orthologs, naked cuticle homolog 1 (NKD1) and 2 (NKD2). NKD1 is located in chromosome16q12.1, and NKD2 is located in chromosome 5p15.3. Loss of heterozygosity has been frequently found in these regions in different types of tumors, including breast cancer [9–13]. Both NKD1 and NKD2 have been reported to antagonize canonical Wnt signaling by interacting with Dishevelled through their EF-hand-like motifs [14]. In addition, NKD2 has been demonstrated to bind to Dishevelled through its TGFα binding region [15]. Human NKD1 and 2 are only 40% identical to each other and they are approximately 70% identical to their respective orthologs in mouse. The C-terminus of NKD2 is highly disordered, while the N-terminal region of NKD2 contains most of the functional domain, which includes myristoylation, an EF-hand motif, a Dishevelled binding region, and a vesicle recognition and membrane targeting motif [15–17]. NKD2 may function as a switch protein through its several functional motifs [14]. The promoter region of NKD2 is hypermethylated in glioblastoma cells [18]. In this study, we studied the epigenetic regulation and function of NKD2 in breast cancer.

## RESULTS

### NKD1 and NKD2 expression are regulated by promoter region methylation in breast cancer cell lines

**Figure 1:**
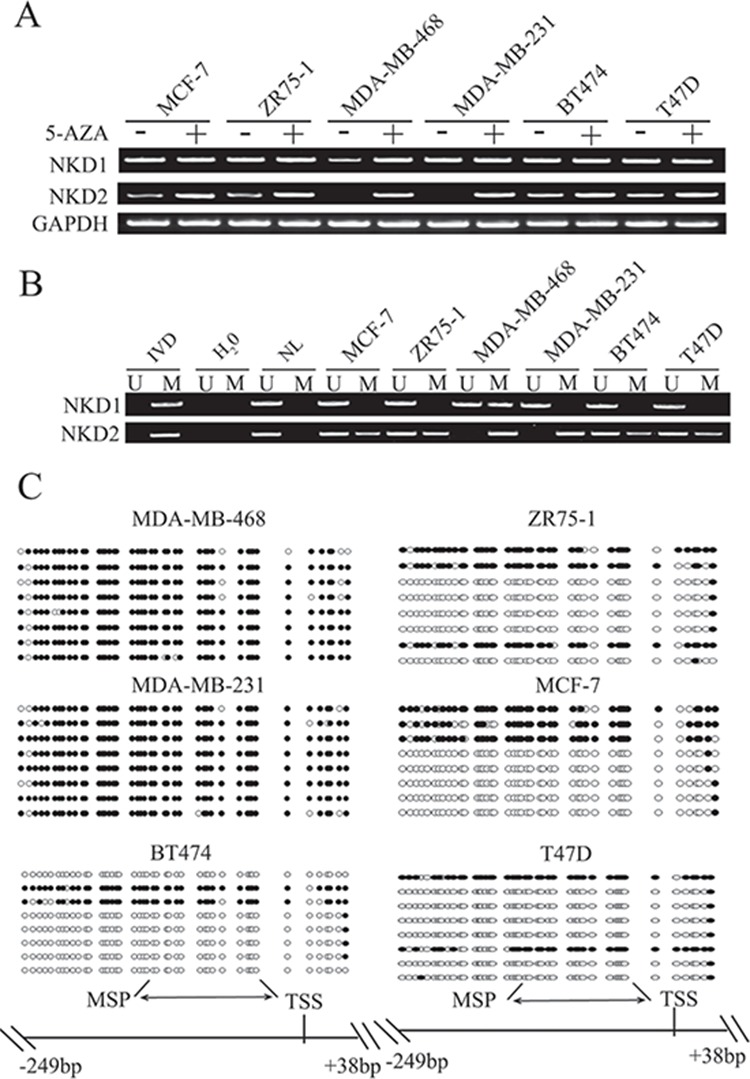
The expression and methylation status of NKD1 and NKD2 in breast cancer cells **A.** NKD1 and NKD2 expression were detected by semi-quantitative RT-PCR. MCF-7, ZR-75-1, MDA-MB-468, MDA-MB-231, BT474, and T47D are breast cancer cell lines. GAPDH: internal control. (−): no 5-AZA treatment; (+): 5-AZA treated. **B.** Methylation status of NKD1 and NKD2 in breast cancer cell lines. IVD: *in vitro* methylated DNA, used as methylated control; NL: normal lymphocyte DNA, used as unmethylated control; U: unmethylated alleles; M: methylated alleles. **C.** Bisulfite sequencing of NKD2 in breast cancer cell lines. Double-headed arrow: MSP PCR product spanning 120–126 bp. Filled circles: methylated CpG sites; open circles: unmethylated CpG sites. TSS: transcriptional start site.

To explore the regulation mechanisms of the NKD gene family in breast cancer, the expression levels of NKD1 and NKD2 were examined by semi-quantitative reverse transcription PCR (RT-PCR). Low level expression of NKD1 was detected in MDA-MB-468. Expression of NKD1 was observed in MCF7, ZR75-1, MDA-MB-231, BT474 and T47D cells. Low level expression of NKD2 was found in MCF7, ZR75-1, BT474 and T47D cells. Loss of NKD2 expression was observed in MDA-MB-231 and MDA-MB-468 cells (Fig. 1A). The promoter region methylation was examined by methylation specific PCR (MSP). NKD1 was partially methylated in MDA-MB-468 and it was unmethylated in MCF7, ZR75-1, MDA-MB-231, BT474 and T47D cells. NKD2 was found to be completely methylated in MDA-MB-231 and MDA-MB-468 cells, and partially methylated in MCF7, ZR75-1, T47D and BT474 cells (Fig 1B). The above results demonstrate that loss or reduction of NKD1 and NKD2 expression is correlated with promoter region hypermethylation in human breast cancer cells. Representative bisulfite sequencing results of NKD2 were shown in Fig. 1C. NKD2 was densely methylated in MDA-MB-231 and MDA-MB-468 cells, and partially methylated in BT474, MCF7, ZR75-1 and T47D cells. These results further validated the efficiency of the MSP primers and the density of promoter region methylation (Fig. 1C). To determine whether NKD1 and NKD2 expression were directly regulated by promoter region methylation, MCF7, ZR75-1, MDA-MB-468, MDA-MB-231, BT474 and T47D cells were treated with the demethylating agent 5-aza-2′-deoxycytidine (5-AZA). Increased expression of NKD1 was observed in MDA-MB-468. No NKD1 expression changes were found in MCF7, ZR75-1, MDA-MB-231, BT474 and T47D cells. Increased expression of NKD2 was observed in MCF7, ZR75-1, BT474 and T47D cells. Re-expression of NKD2 was induced in MDA-MB-468 and MDA-MB-231 (Fig. 1A). These results suggest that the expression of NKD1 and NKD2 is regulated by promoter region methylation in breast cancer cells.

### NKD2 is frequently methylated in primary human breast cancer

**Figure 2:**
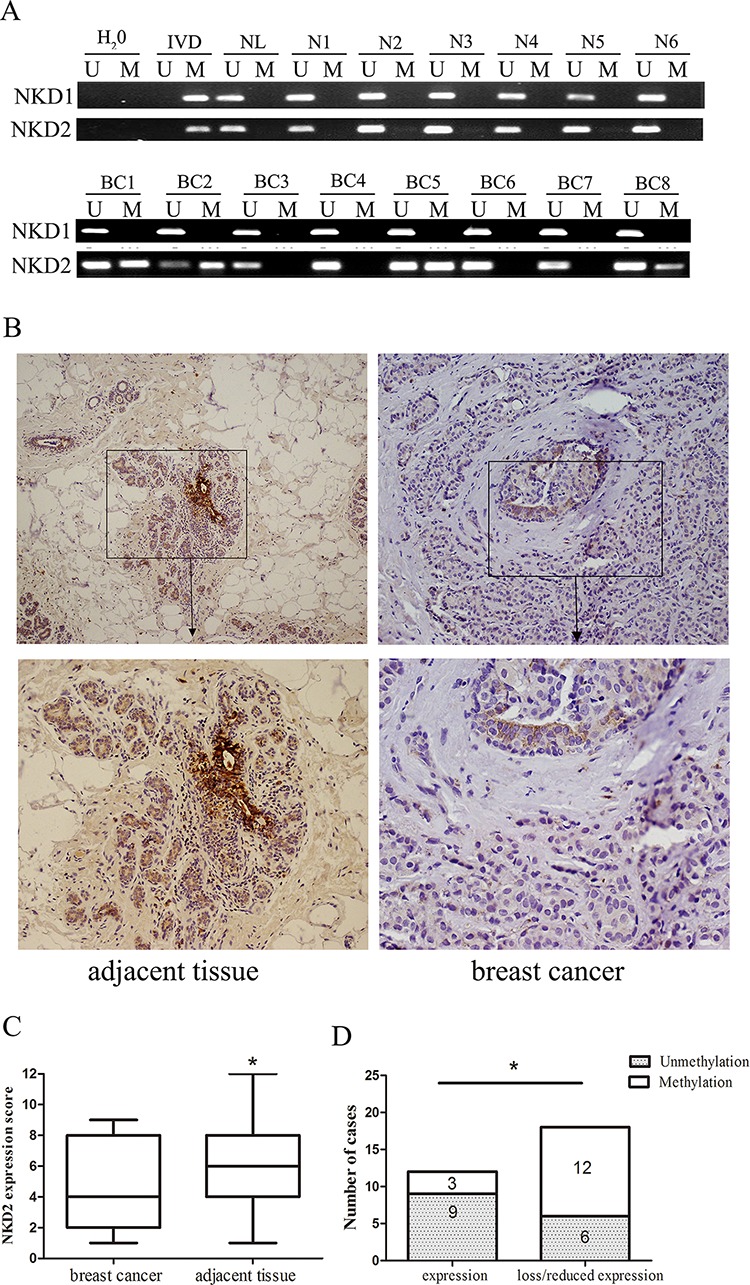
Representative results of NKD1 and NKD2 methylation and expression in primary breast cancer **A.** Representative MSP results of NKD1 and NKD2 in human normal breast tissue samples (N1-N6) and primary breast cancer tissues (BC1-BC8). **B.** Immunohistochemistry (IHC) shows representative NKD2 protein staining in breast cancer and adjacent tissue samples (upper boxes, X200; lower boxes, X400). **C.** NKD2 expression scores are shown as box plots. Horizontal lines represent the median score; the bottom and top of the boxes represent the 25th and 75th percentiles, respectively; vertical bars represent the range of expression. NKD2 expression is significantly different in 30 cases of matched tumor and adjacent tissue samples. *p < 0.05. **D.** The association of NKD2 expression and promoter region hypermethylation was analyzed in 30 cases of matched primary breast cancer and adjacent tissue samples. *p < 0.05.

To further explore the methylation status of NKD1 and NKD2 in primary human breast cancer, 68 cases of primary breast cancer tissues were detected by MSP. Six cases of normal breast tissues from non-cancerous patients were analyzed to rule out tissue specific methylation. NKD1 was unmethylated in 6 normal breast tissue samples and 68 cases of human primary breast cancer samples. The results demonstrate that NKD1 may not play an important role in breast cancer and progression. NKD2 was methylated in 51.4% (35/68) of primary breast cancer samples, and no methylation of NKD2 was detected in normal breast tissue samples (Fig. 2A). The associations between NKD2 methylation and the clinical factors of breast cancer patients are shown in Table 1. NKD2 methylation was significantly associated with tumor stage (*p* < 0.05), but no association was found between NKD2 methylation and age, tumor grade, tumor size, Lymph node metastasis, and the expression of ER, PR, HER2, p53 or Ki-67 (all *p* > 0.05).

**Table 1:** Clinic-pathological features and NKD2 methylation status in breast cancer patients

Clinical parameter		Number (*n* = 68)	Methylation status		*p* value
			Unmethylated *N* = 33(48.6%)	Methylated *N* = 35(51.4%)	
**Age**					
	< 50	31	16	15	0.641
	≥ 50	37	17	20	
**Tumor grade**					
	II	30	12	18	
	II–III	4	3	1	0.209
	III	22	9	13	
	unknown	12	9	3	
**Tumor stage**					
	I–II	58	32	26	0.021*
	III	10	1	9	
**Tumor size**					
	≤ 5cm	60	32	28	0.072
	> 5cm	8	1	7	
**Lymph node metastasis**					
	no	35	19	16	0.328
	yes	33	14	19	
**ER status**					
	Positive	51	22	29	
	Negative	15	9	6	0.173
	unknown	2	2	0	
**PR status**					
	Positive	45	23	22	
	Negative	22	9	13	0.429
	unknown	1	1	0	
**HER2 status**					
	Positive	64	30	34	
	Negative	2	1	1	0.334
	unknown	2	2	0	
**Ki-67**					
	≤ 25%	22	10	12	
	> 25%	44	21	23	0.330
	unknown	2	2	0	
**P53**					
	Positive	44	20	24	
	Negative	19	10	9	0.756
	unknown	5	3	2	

As NKD1 methylation did not appear to be a major event in human breast cancer, we mainly focused on the mechanisms of NKD2 in breast carcinogenesis. The expression of NKD2 was evaluated by immunohistochemistry (IHC) in 30 cases of available matched breast cancer and adjacent tissue samples. NKD2 staining was observed in the cytoplasm of the adjacent tissue samples and its expression was significantly reduced in primary breast cancer samples (Fig. 2B and 2C). The reduction in NKD2 expression was associated with its promoter region hypermethylation (*p* < 0.05; Fig. 2D). These results suggest that NKD2 expression may be regulated by promoter region methylation in primary breast cancer.

### The cell invasion and migration were no affected by NKD2 in breast cancer cells

The transwell assay was employed to evaluate the effect of NKD2 on cell invasion in MDA-MB-468 and MDA-MB-231 cells. The number of invasive cells for each high power field under the microscope was 503 ± 22.5 vs. 481 ± 14.7 in MDA-MB-468 cells and 436.3 ± 11.5 vs. 429 ± 14.7 cells before and after restoration of NKD2 expression. The cell number was not significantly different before and after re-expression of NKD2 in MDA-MB-468 and MDA-MB-231 cells (all *P* > 0.05, Supplementary Figure. 1A).

Next, the transwell assay in the absence of ECM gel (extracellular matrix gel) coating was employed to explore the effect of NKD2 on cell migration in MDA-MB-468 and MDA-MB-231 cells. The number of migrated cells for each high power field under the microscope was 880 ± 20 vs. 863.3 ± 15.3 in MDA-MB-468 cells and 816 ± 31.7 vs. 794.7 ± 14.0 in MDA-MB-231 cells before and after restoration of NKD2 expression. The cell number was not significantly different before and after re-expression of NKD2 (all *P* > 0.05, Supplementary Figure. 1B).

### Restoration of NKD2 expression suppresses cell proliferation and induces G1/S arrest in breast cancer

**Figure 3:**
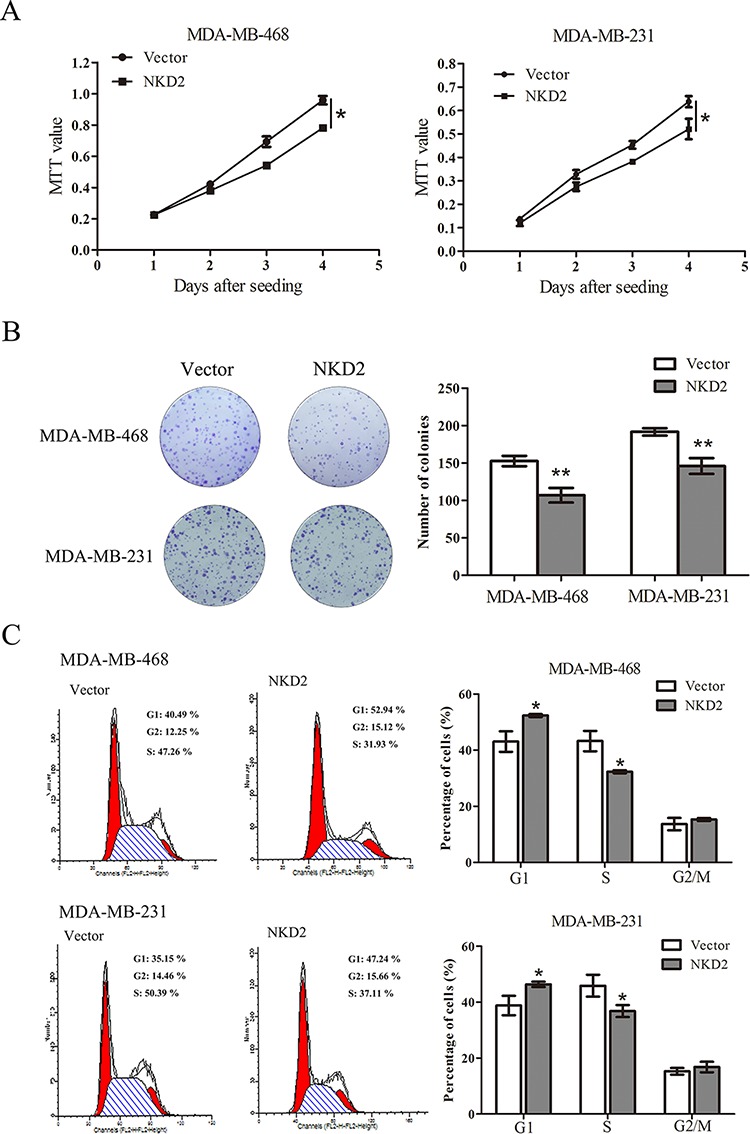
NKD2 suppresses breast cancer cell proliferation **A.** Growth curves represent the effect of NKD2 on cell proliferation in NKD2 re-expressed and unexpressed MDA-MB-468 and MDA-MB-231 cell lines. *p < 0.05. **B.** Representative results of colony formation in NKD2 re-expressed and unexpressed MDA-MB-468 and MDA-MB-231 cell lines. **p < 0.01. **C.** Flow cytometry results show the cell phase distribution in NKD2 unexpressed and re-expressed MDA-MB-468 and MDA-MB-231 cells. *p < 0.05.

To evaluate the effect of NKD2 on breast cancer carcinogenesis, cell viability and colony formation assays were performed in cell lines with no NKD2 expression and stably transfected cell lines in which NKD2 was re-expressed. Re-expression of NKD2 reduced cell proliferation (*p* < 0.05, Fig. 3A) and colony formation (*p* < 0.01, Fig. 3B) in MDA-MB-468 and MDA-MB-231 cells. These results indicate that NKD2 inhibits breast cancer cell proliferation. To further understand the mechanism of NKD2 in breast cancer development, cell cycle analysis was performed by flow cytometry. In MDA-MB-468 cells, the cell phase distributions before and after re-expression of NKD2 were as follows: G0/1 phase: 43.08 ± 3.64% vs. 52.35 ± 0.51%, S phase: 43.26 ± 3.61% vs. 32.32 ± 0.56%, and G2/M phase: 13.66 ± 2.2% vs. 15.32 ± 0.53%. In MDA-MB-231, the cell phase distributions before and after re-expression of NKD2 were as follows: G0/1 phase: 38.81 ± 3.48% vs. 46.37 ± 0.94%, S phase: 45.89 ± 3.9% vs. 36.82 ± 2.2%, and G2/M phase: 15.3 ± 1.2% vs. 16.81 ± 1.9% (Fig. 3C). The percentage of cells in S phase was significantly reduced (*p* < 0.05) after NKD2 re-expression, and the percentage of cells in G0/1 phase was significantly increased (*p* < 0.05) after NKD2 re-expression. These results suggest that NKD2 induced G1/S arrest.

### NKD2 inhibits Wnt/β-catenin signaling in breast cancer

**Figure 4:**
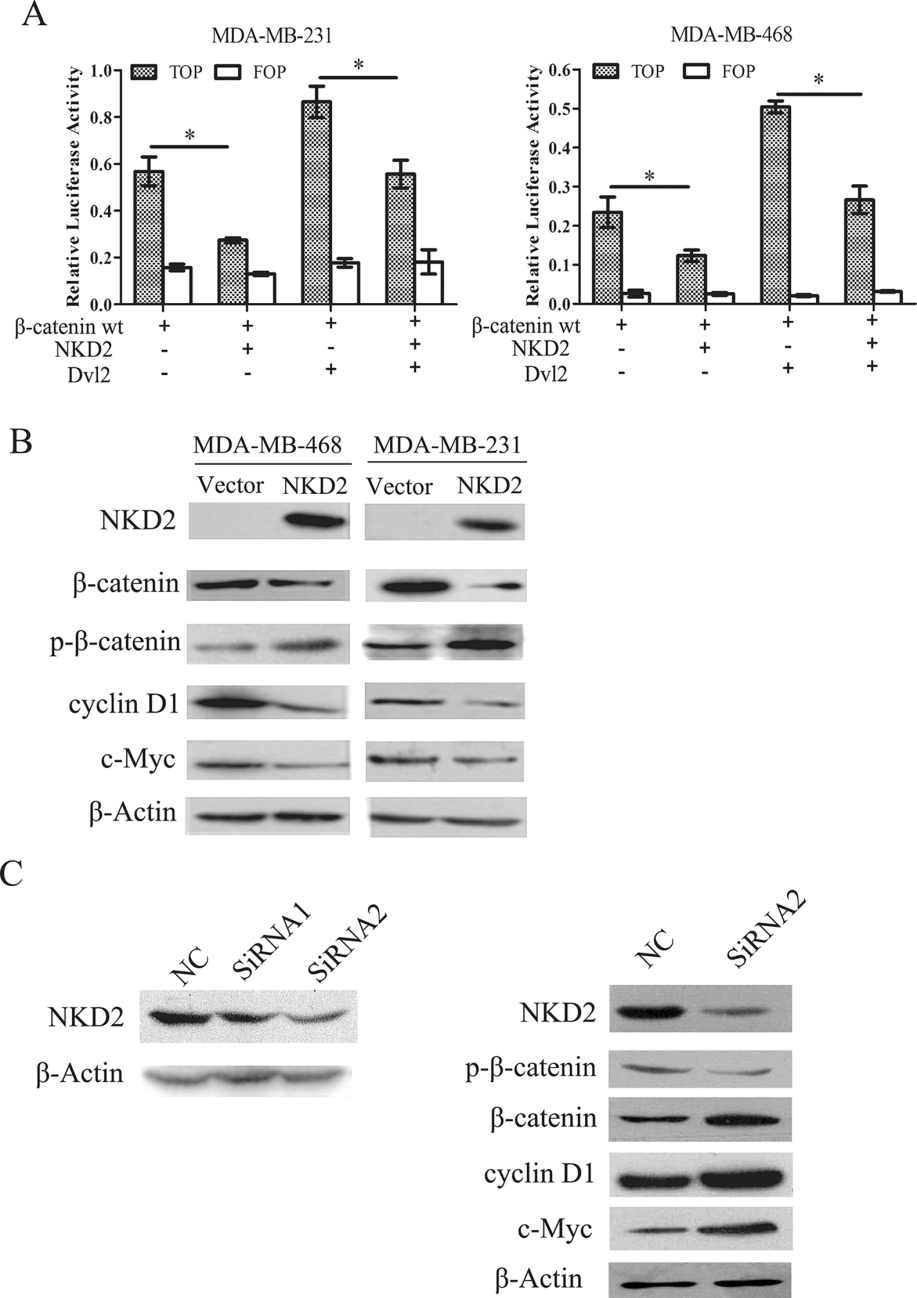
NKD2 inhibits canonical Wnt signaling in human breast cancer cells **A.** Results of TCF/LEF luciferase reporter assay. Wild type β-catenin expression vector was co-transfected with TCF/LEF Topflash or Fopflash reporter in MDA-MB-468 and MDA-MB-231 cells. The graphs show the luciferase activity in MDA-MB-468 and MDA-MB-231 co-transfected with Topflash + β-wt, Topflash + β-wt + NKD2, Topflash + β-wt + DVL2 and Topflash + β-wt + DVL2 + NKD2.*p < 0.05. **B.** NKD2 regulates the downstream genes of Wnt signaling. The expression levels of β-catenin, cyclin D1 and c-myc decreased and the level of phosphorylated β-catenin (p-β-catenin) increased after re-expression of NKD2 in MDA-MB-468 and MDA-MB-231 cells. **C.** NKD2 knockdown induces activation of Wnt signaling. The level of p-β-catenin decreased and the expression of β-catenin, c-myc and cyclin D1 increased after knockdown of NKD2 in BT474 cells.

During activation of Wnt signaling, β-catenin accumulates in the cytoplasm and is then translocated into the nucleus where it activates the transcription of Wnt target genes, such as cyclin D1 and c-myc [19–21]. NKD2 has been reported to negatively regulate canonical Wnt signaling by binding to Dishevelled (Dvl) in HEK293 cells [22]. To determine whether the canonical Wnt signaling pathway is regulated by NKD2 in human breast cancer, a dual-luciferase reporter assay was employed. The activity of TCF/LEF was significantly inhibited by co-transfection of wild-type β-catenin and NKD2. The activity of TCF/LEF was increased by co-transfecting Dvl2 and wild-type β-catenin, but it was decreased after co-transfecting NKD2, Dvl2, and wild-type β-catenin (Fig. 4A). Western blotting indicated that the level of β-catenin was reduced and the level of phospho-β-catenin increased. Expression of the c-myc and cyclin D1 target genes was reduced after re-expression of NKD2 in MDA-MB-468 and MDA-MB-231 cells (Fig. 4B). To further validate the effect of NKD2 on Wnt signaling, siRNA knockdown technique was employed. After knockdown of NKD2 in BT474 cells, the expression of β-catenin, c-myc and cyclin D1 increased, and the level of phospho-β-catenin was reduced (Fig. 4C). These results suggest that NKD2 represses canonical Wnt signaling in human breast cancer cells.

### NKD2 suppresses breast cancer growth in xenograft mice

**Figure 5:**
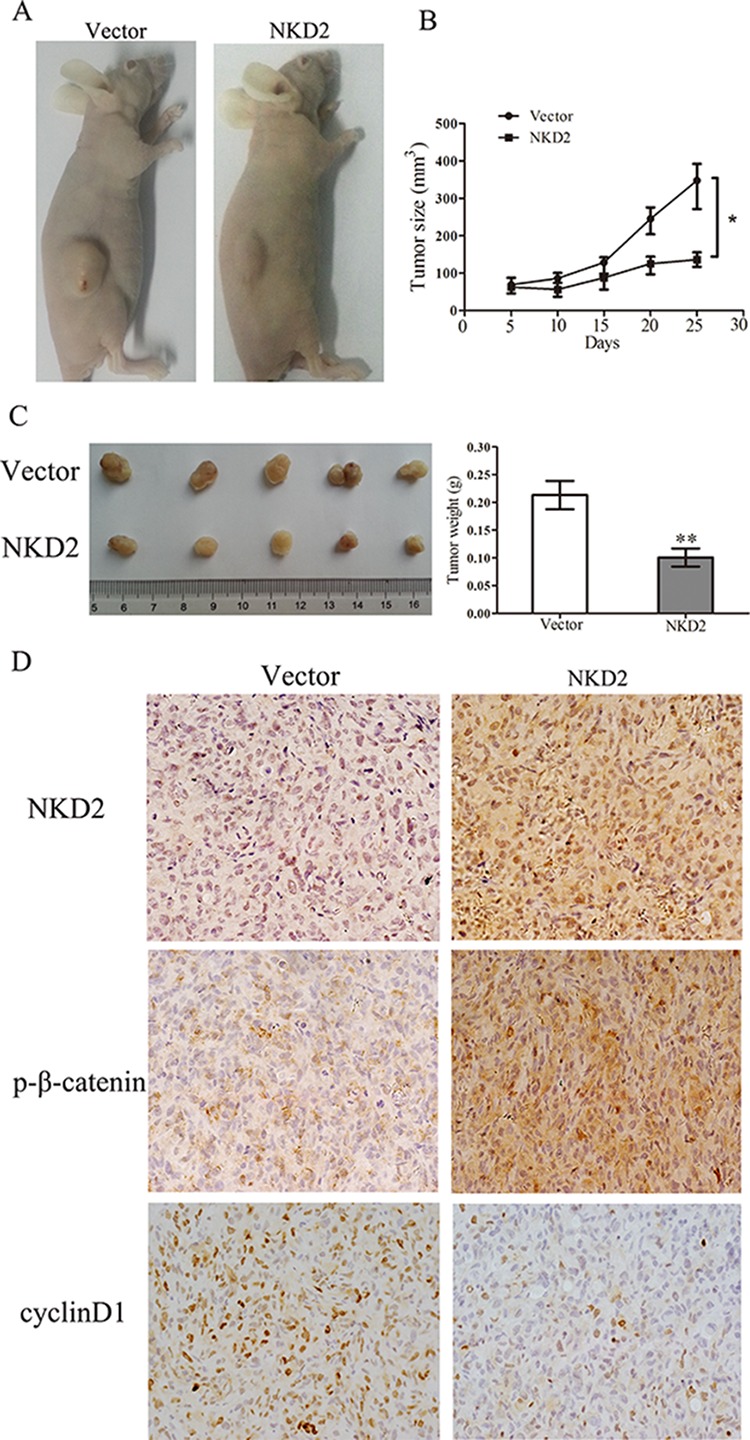
NKD2 suppresses tumor growth in xenograft mice **A.** Representative results of xenograft tumors in nude mice for NKD2 expressed and unexpressed MDA-MB-231 cells. **B.** Subcutaneous tumor growth curves for xenograft mice in NKD2 unexpressed and re-expressed groups at different times. *p < 0.05. **C.** Tumor weight in nude mice was measured at the 21 days after injection of NKD2 unexpressed and re-expressed MDA-MB-231 cells. **p < 0.01. **D.** Representative photographs of immunohistochemistry (IHC) analysis of NKD2, p-β-catenin and cyclin D1 in xenografts. Staining of NKD2 and p-β-catenin was found in NKD2 re-expressed MDA-MB-231 cell xenografts and the staining of cyclin D1 was reduced. Magnification: 400 ×.

To further explore the effects of NKD2 on breast cancer cell growth, we employed a MDA-MB-231 xenograft mouse model in which NKD2 was either stably expressed or unexpressed. The tumor volume was smaller in NKD2 expressed MDA-MB-231 cell xenograft mice compared to the NKD2 unexpressed group (140.89 ± 18.41 mm^3^ vs. 334.77 ± 49.88 mm^3^, *p* < 0.05). The tumor weight was less in the NKD2 expressed group compared to the unexpressed group (101.75 ± 34.20 mg vs. 213.13 ± 51.15 mg, *p* < 0.01, Fig. 5A–5C). IHC staining demonstrated increased levels of NKD2 and phospho-β-catenin and reduced staining of cyclin D1 in NKD2 re-expressed xenografts (Fig. 5D). These results further suggest that NKD2 suppresses breast cancer growth by inhibiting Wnt signaling.

## DISCUSSION

Accumulation of aberrant genetic and epigenetic changes is regarded to play an important role in many types of human cancers, including breast cancer [23–25]. Many chromosome changes were found in breast cancer, including 1p, 1q, 4p, 4q, 5p12-14, 6q, 8p, 8q, 9p, 9q, 11q13-14, 13q, 16p, 16q, 17p, 17q12, 17q22-24, 18p, 19p, 19q, 20q13, 21q and Xq [26]. In breast cancer, 5–10% is caused by germ-line mutations in well identified breast cancer susceptibility genes. The high-risk breast cancer susceptibility genes including BRCA1, BRCA2, PTEN, TP53, LKB1/SYK11 and CDH1, with relative lifetime risks higher than 4. The CHEK2, TGFβ1, CASP8 and ATM genes belong to the ‘low to moderate-risk’ breast cancer susceptibility genes [27]. In sporadic breast cancers, germline BRCA1 mutations are not detected, but somatic inactivation of the BRCA1 gene by DNA hypermethylation has been reported to occur as an epigenetic event [26]. NKD2 has been reported to be frequently methylated in human gliomas [18]. Recent study suggested that NKD2 is a tumor suppressor in osteosarcoma [28]. Our study demonstrated that NKD2 was frequently methylated in human breast cancer and its expression was regulated by promoter region methylation. These results suggest that aberrant methylation of NKD2 may involve in breast cancer carcinogenesis and progression. Methylation of NKD2 was associated with tumor stage. It indicates that NKD2 methylation may serve as a breast cancer prognostic marker. In previous report, Feng et al. found NKD2 is methylated in about 10% of breast cancer [29]. Our study found NKD2 is methylated in 51.4% of breast cancer. This discrepancy was generated by different detection method. MSP is very specific, sensitive and reproductive. The result of bisulfite pyrosequencing is depending on the cut off value. Re-expression of NKD2 suppressed cell proliferation and induced G1/S arrest in MDA-MB-468 and MDA-MB-231 cells. The results suggest that NKD2 is a tumor suppressor in human breast cancer. NKDs have been shown to negatively regulate canonical Wnt signaling through binding to Dvl [17, 16, 30]. The phosphoprotein Dishevelled is a multifunctional protein harboring docking sites for more than 20 suspected binding partners. Prominent domains found in Dishevelled, such as DIX, PDZ and DEP, are highly conserved from the single fly Dsh to the mammalian Dvl1, Dvl2 and Dvl3. The expression of Dvl2 accounts for more than 95% of the total pool of Dvls, whereas Dvl1 and Dvl3 each account for less than 2% in both mouse and mammalian cells [31]. In our dual-luciferase reporter assay, the activity of TCF/LEF was inhibited by NKD2. TCF/LEF activity increased when co-transfecting Dvl2 and wild-type β-catenin, and the activity decreased by co-transfecting NKD2 with Dvl2 and wild-type β-catenin. These results suggest that NKD2 inhibits Wnt signaling by interacting with Dvl2 in human breast cancer cells. The specific interaction between NKD2 and Dvl1 or Dvl3 remains to be determined. Through our *in vitro* and *in vivo* studies, it was validated that NKD2 suppresses breast cancer cell growth by inhibiting Wnt signaling.

In conclusion, NKD2 is frequently methylated in human breast cancer and its expression is regulated by promoter region methylation. Methylation of NKD2 is associated with tumor stage. NKD2 methylation may serve as a breast cancer diagnostic and prognostic marker. NKD2 suppresses breast cancer cell growth by inhibiting Wnt signaling both *in vitro* and *in vivo*.

## MATERIALS AND METHODS

### Human breast cancer tissues and cell lines

A total of 68 cases of primary breast cancer were collected at the Chinese PLA General Hospital from 2012 to 2014, and tumor staging was determined according to the American Joint Committee on Cancer (AJCC) Cancer Staging Manual, 2010 (7th edition). Thirty cases of paraffin blocks were available with matched adjacent tissue samples. The clinic-pathological factors are shown in table 1. Six cases of normal breast tissues were collected from non-cancerous patients in the Chinese PLA General Hospital and stored as fresh frozen samples. All samples were collected under the approved guidelines of the Chinese PLA General Hospital's institutional review board. Six breast cancer cell lines (MCF-7, ZR75-1, MDA-MB-468, MDA-MB-231, T47D and BT474) were previously established from primary breast cancer and maintained in RPMI-1640 (Intrivogen, Carlsbad, CA, USA) supplemented with 10% fetal bovine serum (Hyclone, Logan, UT) and 1% penicillin and streptomycin in a 5% CO_2_ incubator at 37°C.

### 5-aza-2′-deoxycytidine treatments, RNA Isolation and RT-PCR

Breast cancer cell lines were split to a low density (30% confluence) 12 hours before treatment. Cells were treated with 5-AZA (Sigma, St. Louis, MO, USA) at a concentration of 2 μM, and the growth medium was changed every 24 hours for a total of 96 hours of treatment. Total RNA was extracted by using Trizol reagent (Invitrogen, Carlsbad, CA). The cDNA synthesis and semi-quantitative RT-PCR were performed as described previously [32]. GAPDH was used as an internal control.

### Bisulfite modification, MSP and bisulfite sequencing

Genomic DNA from breast cancer cell lines and breast cancer tissue samples were prepared by the proteinase-K method. MSP and bisulfite sequencing were performed as described previously [33, 34]. MSP primers were designed according to genomic sequences around the transcription start site in the CpG island of NKD1 and NKD2 genes to detect unmethylated (U) and methylated (M) alleles. The primers were oligo-synthesized (BGI, Beijing, China). The size of the unmethylated PCR product is 126bp and the methylated PCR product is 120bp. All primers are listed in Table S1.

### Immunohistochemistry staining

Immunohistochemistry (IHC) was performed in primary cancer and paired adjacent tissue samples. The procedure was performed as described previously [32]. NKD2 antibody (Novus Biology, CO, USA) was used at a 1/500 dilution overnight at 4°C. Anti- phospho-β-catenin (Bioworld Technology, MN, USA) and anti-cyclin D1 (Bioworld Technology, MN, USA) at a 1/150 dilution were incubated overnight at 4°C. The staining intensity and extent of the staining area were scored using the German semi-quantitative scoring system as previously described [32].

### Plasmid construction

Human full-length NKD2 CDS was amplified from normal human gastric mucosa and then subcloned into the pLenti6-GFP lentivirus expression vector. The primers were 5′-GAGGATCCGCCA CCATGGGGAAACTGCAGTCGAAG-3′ (F) and 5′-GATCTCGAGCTAGGACGGGTGGAAGTGGT-3′(R). NKD2 expressing lentiviral or empty vectors were packaged using the ViraPower™ lentiviral expression system (Invitrogen, San Diego, CA, USA). Lentivirus was added to the growing medium of MDA-MB-468 and MDA-MB-231 cells, and NKD2 stably expressed cells were selected by blasticidin (2.5 μg/ml, Invitrogen).

### Cell viability assay

NKD2 unexpressed and stably expressed cells were seeded into 96-well plates (2 × 10^3^ cells/well), and the cell viability was measured daily for 96 hours using the MTT (3-(4, 5-dimethylthiazol-2-yl)-2, 5-diphenyltetrazolium bromide) Kit (KeyGEN Biotech, Nanjing, China) according to the manufacturer's instructions. The data were plotted as means ± SD.

### Colony formation assay

NKD2 unexpressed and stably expressed cells were seeded at 500 cells per well in 6-well culture plates in triplicate. The complete growth medium conditioned with blasticidin at 2.5ug/ml was exchanged every 72 hours. After 2 weeks, cells were fixed with 75% ethanol for 30 min and stained with 0.2% crystal violet (Beyotime, Nanjing, China) for visualization and counting.

### Flow cytometry analysis

For cell cycle analysis, NKD2 unexpressed and stably expressed cells were starved 12 hours for synchronization, and the cells were re-stimulated with 10% FBS for 24 hours. Cells were fixed with 70% ethanol and treated by Cell Cycle Detection Kit (KeyGen Biotech, Nanjing, China) according to the manufacturer's instructions. The cells were then sorted by a FACS Caliber flow cytometer (BD Biosciences, Mansfield, CA). The cell phase distribution was analyzed by the Modfit software.

### Dual-Luciferase reporter assay

MDA-MB-468 and MDA-MB-231 cells were seeded at 7 × 10^3^ cells per well in 96-well culture plates 24 hours before transfection. Cells were transiently transfected using Lipofectamine 2000 (Invitrogen, San Diego, CA) with an appropriate combination of the reporter, expression plasmids and control vector, including 30 ng/well TOPflash reporter vector (TCF/LEF–responsive reporter), 3ng/well pRL-TK as an internal control reporter, and 50ng/well pCI-neo-β-catenin, which expressed wild-type β-catenin and was used to activate the reporter gene. As a negative control, another group of MDA-MB-231and MDA-MB-468 cells were transfected with 30ng/well Fopflash reporter vector, 3ng/well pRL-TK control vector and wild-type β-catenin vector (50 ng/well). Increasing amounts of 60 ng/well NKD2 vector and 50 ng/well Dvl2 vector were then transfected into cells with the Topflash reporter vector, pRL-TK control vector and pCI-neo-β-catenin to evaluate the regulatory function of NKD2 in the Wnt signaling. Forty-eight hours after transfection, relative luciferase activities were measured with the Dual Luciferase Reporter Assay system (Promega, Shanghai, China) according to the manufacturer's protocol. For each experiment, the luciferase reporter assay was performed three times.

### siRNA knockdown

Two selected siRNAs targeting NKD2 and a RNAi Negative Control Duplex were used in this study. The sequences were as follows: siRNA duplex 1 (sense 5′-CACGCUCUAUGACUUUGACTT-3′ and antisense 5′-GUCAAAGUCAUAGAGCGUGTT-3′); siRNA duplex 2 (sense 5′-GGGAUUGAGAACUACACGUTT-3′ and antisense 5′-ACGUGUAGUUCUCAAUCCCTT-3′); RNAi Negative Control Duplex (sense 5′-UUCUCCGAACGUGUCACGUTT-3′ and antisense 5′-ACGUGACACGUUCGGAGAATT-3′). The RNAi oligonucleotide or RNAi negative control duplex (Gene Pharma Co, Shanghai, China) were transfected into BT474 cells using Lipofectamine RNAiMAX Reagent (Invitrogen, San Diego, CA) according to the manufacturer's instructions. The siRNA duplex 2 was more effective at knocking down NKD2 than the siRNA duplex 1.

### Protein preparation and western blotting

Protein preparation and western blotting were performed as described previously [32]. The antibodies for immune blot analysis were as follows: rabbit anti-NKD2 (Cell Signaling Technology, Danvers, MA), rabbit cyclin D1 (Bioworld Technology, MN, USA), rabbit c-myc (Bioworld Technology, MN, USA), phospho-β-catenin (Bioworld Technology, MN, USA), β-actin (Bioworld Technology, MN, USA) and β-catenin (Epitomics, Burlingame, CA). The data were normalized to β-actin.

### *In vivo* tumorigenicity

NKD2 stably expressed and unexpressed MDA-MB-231 cells (4 × 10^6^ cells in 0.2 ml phosphate-buffered saline) were subcutaneously injected into the dorsal flank of 5-week-old female BALB/c nude mice. The tumor size was measured every 5 days for 4 weeks beginning 5 days after implantation. The tumor volume was calculated according to the following formula: V = L × W^2^/2, where V, volume (mm^3^); L, biggest diameter (mm); W, smallest diameter (mm). All procedures were approved by the Animal Ethics Committee of the Chinese PLA General Hospital.

### Transwell assay

NKD2 unexpressed and re-expressed MDA-MB-468 and MDA-MB-231 cells were suspended in serum-free medium. Cells (5 × 10^4^) were placed into the upper chamber of an 8 μm pore size Transwell apparatus (Corning, NY, USA) and incubated for 12 hours. Cells that migrated to the lower surface of the membrane were stained with crystal violet and counted in three independent high-power fields (×200). For invasion analysis, NKD2 unexpressed and re-expressed MDA-MB-468 and MDA-MB-231 cells (1 × 10^5^) were seeded into the upper chamber of a transwell apparatus coated with Matrigel (BD Biosciences, San Jose, CA) and incubated for 24 hours. Cells that invaded into the lower membrane surface were stained with crystal violet and counted in three independent high-power fields (×200).

### Statistical analysis

SPSS 17.0 software was used for data analysis. All data were presented as means ± standard deviation (SD) of at least three independent experiments and analyzed using the Student's *t* test. The Chi-squared test was used to analyze the association of NKD2 methylation status with clinic-pathologic factors and the association of NKD2 expression with methylation status. The value of *p* < 0.05 was considered to be statistically significant.

## SUPPLEMENTARY FIGURE AND TABLE



## References

[1] Zob D, Vasilescu M, Gruia M, Anghel R (2013). Breast cancer. Screening criteria.

[2] Azim HA, d Ibrahim AS (2014). Breast cancer in Egypt, China and Chinese: statistics and beyond. Journal of thoracic disease.

[3] Huang Y, Nayak S, Jankowitz R, Davidson NE, Oesterreich S (2011). Epigenetics in breast cancer: what's new?. Breast cancer research: BCR.

[4] King TD, Suto MJ, Li Y (2012). The Wnt/beta-catenin signaling pathway: a potential therapeutic target in the treatment of triple negative breast cancer. Journal of cellular biochemistry.

[5] Guo J, Cagatay T, Zhou G, Chan CC, Blythe S, Suyama K, Zheng L, Pan K, Qian C, Hamelin R, Thibodeau SN, Klein PS, Wharton KA (2009). Mutations in the human naked cuticle homolog NKD1 found in colorectal cancer alter Wnt/Dvl/beta-catenin signaling. PloS one.

[6] Suzuki H, Toyota M, Carraway H, Gabrielson E, Ohmura T, Fujikane T, Nishikawa N, Sogabe Y, Nojima M, Sonoda T, Mori M, Hirata K, Imai K (2008). Frequent epigenetic inactivation of Wnt antagonist genes in breast cancer. British journal of cancer.

[7] Jonsson M, Borg A, Nilbert M, Andersson T (2000). Involvement of adenomatous polyposis coli (APC)/beta-catenin signalling in human breast cancer. European journal of cancer. (Oxford, England: 1990).

[8] Geyer FC, Lacroix-Triki M, Savage K, Arnedos M, Lambros MB, MacKay A, Natrajan R, Reis-Filho JS (2010). β-Catenin pathway activation in breast cancer is associated with triple-negative phenotype but not with CTNNB1 mutation. Modern Pathology.

[9] Friedrich K, Weber T, Scheithauer J, Meyer W, Haroske G, Kunze KD, Baretton G (2008). Chromosomal genotype in breast cancer progression: comparison of primary and secondary manifestations. Cellular oncology : the official journal of the International Society for Cellular Oncology.

[10] Frye M, Dragoni I, Chin SF, Spiteri I, Kurowski A, Provenzano E, Green A, Ellis IO, Grimmer D, Teschendorff A, Zouboulis CC, Caldas C, Watt FM (2010). Genomic gain of 5p15 leads to over-expression of Misu (NSUN2) in breast cancer. Cancer letters.

[11] Focken T, Steinemann D, Skawran B, Hofmann W, Ahrens P, Arnold N, Kroll P, Kreipe H, Schlegelberger B, Gadzicki D (2011). Human BRCA1-associated breast cancer: no increase in numerical chromosomal instability compared to sporadic tumors. Cytogenetic and genome research.

[12] Argos M, Kibriya MG, Jasmine F, Olopade OI, Su T, Hibshoosh H, Ahsan H (2008). Genomewide scan for loss of heterozygosity and chromosomal amplification in breast carcinoma using single-nucleotide polymorphism arrays. Cancer Genetics and Cytogenetics.

[13] Sheu JC, Lin YW, Chou HC, Huang GT, Lee HS, Lin YH, Huang SY, Chen CH, Wang JT, Lee PH, Lin JT, Lu FJ, Chen DS (1999). Loss of heterozygosity and microsatellite instability in hepatocellular carcinoma in Taiwan. British journal of cancer.

[14] Hu T, Krezel AM, Li C, Coffey RJ (2006). Structural studies of human Naked2: a biologically active intrinsically unstructured protein. Biochemical and biophysical research communications.

[15] Li C, Franklin JL, Graves-Deal R, Jerome WG, Cao Z, Coffey RJ (2004). Myristoylated Naked2 escorts transforming growth factor alpha to the basolateral plasma membrane of polarized epithelial cells. Proceedings of the National Academy of Sciences of the United States of America.

[16] Rousset R, Mack JA, Wharton KA, Axelrod JD, Cadigan KM, Fish MP, Nusse R, Scott MP (2001). Naked cuticle targets dishevelled to antagonize Wnt signal transduction. Genes & development.

[17] Zeng W, Wharton KA, Mack JA, Wang K, Gadbaw M, Suyama K, Klein PS, Scott MP (2000). naked cuticle encodes an inducible antagonist of Wnt signalling. Nature.

[18] Gotze S, Wolter M, Reifenberger G, Muller O, Sievers S (2010). Frequent promoter hypermethylation of Wnt pathway inhibitor genes in malignant astrocytic gliomas. International journal of cancer Journal international du cancer.

[19] Usongo M, Li X, Farookhi R (2013). Activation of the canonical WNT signaling pathway promotes ovarian surface epithelial proliferation without inducing beta-catenin/Tcf-mediated reporter expression. Developmental dynamics: an official publication of the American Association of Anatomists.

[20] Wang K, Li N, Yeung CH, Li JY, Wang HY, Cooper TG (2013). Oncogenic Wnt/beta-catenin signalling pathways in the cancer-resistant epididymis have implications for cancer research. Molecular human reproduction.

[21] Chen HJ, Hsu LS, Shia YT, Lin MW, Lin CM (2012). The beta-catenin/TCF complex as a novel target of resveratrol in the Wnt/beta-catenin signaling pathway. Biochemical pharmacology.

[22] Hu T, Li C, Cao Z, Van Raay TJ, Smith JG, Willert K, Solnica-Krezel L, Coffey RJ (2010). Myristoylated Naked2 antagonizes Wnt-beta-catenin activity by degrading Dishevelled-1 at the plasma membrane. The Journal of biological chemistry.

[23] Fearon ER, Vogelstein B (1990). A genetic model for colorectal tumorigenesis. Cell.

[24] Guo M, Ren J, Brock MV, Herman JG, Carraway HE (2008). Promoter methylation of HIN-1 in the progression to esophageal squamous cancer. Epigenetics: official journal of the DNA Methylation Society.

[25] Guo M, Ren J, House MG, Qi Y, Brock MV, Herman JG (2006). Accumulation of promoter methylation suggests epigenetic progression in squamous cell carcinoma of the esophagus. Clinical cancer research: an official journal of the American Association for Cancer Research.

[26] Tsuda H (2009). Gene and chromosomal alterations in sporadic breast cancer: correlation with histopathological features and implications for genesis and progression. Breast cancer. Tokyo, Japan.

[27] Oldenburg RA, Meijers-Heijboer H, Cornelisse CJ, Devilee P (2007). Genetic susceptibility for breast cancer: how many more genes to be found?. Critical reviews in oncology/hematology.

[28] Zhao S, Kurenbekova L, Gao Y, Roos A, Creighton CJ, Rao P, Hicks J, Man TK, Lau C, Brown AM, Jones SN, Lazar AJ, Ingram D (2015). NKD2, a negative regulator of Wnt signaling, suppresses tumor growth and metastasis in osteosarcoma. Oncogene.

[29] Feng W, Shen L, Wen S, Rosen DG, Jelinek J, Hu X, Huan S, Huang M, Liu J, Sahin AA, Hunt KK, Bast RC, Shen Y (2007). Correlation between CpG methylation profiles and hormone receptor status in breast cancers. Breast cancer research : BCR.

[30] Yan D, Wallingford JB, Sun TQ, Nelson AM, Sakanaka C, Reinhard C, Harland RM, Fantl WJ, Williams LT (2001). Cell autonomous regulation of multiple Dishevelled-dependent pathways by mammalian Nkd. Proceedings of the National Academy of Sciences of the United States of America.

[31] Lee YN, Gao Y, Wang HY (2008). Differential mediation of the Wnt canonical pathway by mammalian Dishevelleds-1, -2, and -3. Cellular signalling.

[32] Yan W, Wu K, Herman JG, Brock MV, Fuks F, Yang L, Zhu H, Li Y, Yang Y, Guo M (2013). Epigenetic regulation of DACH1, a novel Wnt signaling component in colorectal cancer. Epigenetics: official journal of the DNA Methylation Society.

[33] Herman JG, Graff JR, Myohanen S, Nelkin BD, Baylin SB (1996). Methylation-specific PCR: a novel PCR assay for methylation status of CpG islands. Proceedings of the National Academy of Sciences of the United States of America.

[34] Jia Y, Yang Y, Liu S, Herman JG, Lu F, Guo M (2010). SOX17 antagonizes WNT/beta-catenin signaling pathway in hepatocellular carcinoma. Epigenetics : official journal of the DNA Methylation Society.

