# Supplementary material for: Epigenetic silencing of NKD2, a major component of Wnt signaling, promotes breast cancer growth
Source: Oncotarget. 2015 Jun 19;6(26):22126–38. doi: 10.18632/oncotarget.4244 (PMC4673151; doi:10.18632/oncotarget.4244)
Supplement: Supplementary file 1 [file oncotarget-06-22126-s001.pdf]

SUPPLEMENTARY FIGURE AND TABLE

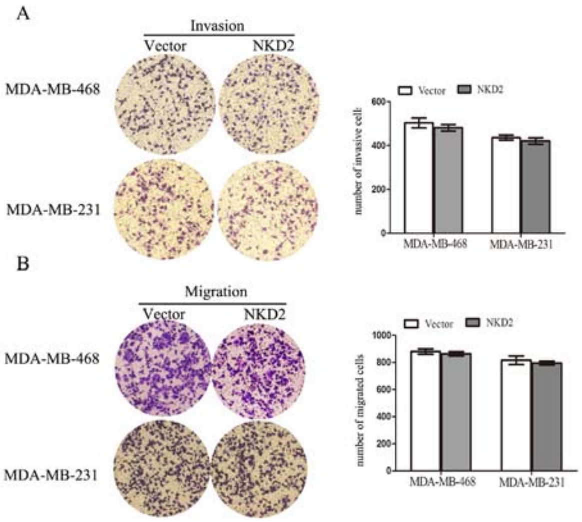

Supplementary Figure S1: The effect of NKD2 in cell migration and invasion. A. Cell invasion in NKD2 unexpressed and expressed MDA-MB-468 and MDA-MB-231 cells. The number of invasive cells is presented by bar diagram. Each experiment was repeated for three times, all $P > 0.05$. **B.** Cell migration in NKD2 unexpressed and expressed MDA-MB-468 and MDA-MB-231 cells. The number of migratory cells is presented by bar diagram. Each experiment was repeated for three times, all $P > 0.05$.

Supplementary Table S1. RT-PCR and MSP primer sequences

**RT-PCR primers**
NKD1- Forward: 5′-AACCACTACTTAGATCTCGCCG-3′<br>NKD1- Reverse: 5′-GAGCCGTTGCTGGAGCTCTG-3′<br>NKD2- Forward: 5′-ACAGGAGGTTGTCTGCACACG-3′<br>NKD2- Reverse: 5′-GACTTGAGGAACTGCTTCTCCG-3′<br>GAPDH- Forward: 5′-GACCACAGTCCATGCCATCAC-3′<br>GAPDH- Reverse: 5′-GTCCACCACCCTGTTGCTGTA-3′
**MSP primers**
NKD1-M- Forward: 5′-GTTCGGCGTTTTCGGGCGTTAGTC-3′<br>NKD1-U- Forward: 5′-GGGTTTGGTGTTTTTGGGTGTTAGTT-3′<br>NKD1-M- Reverse: 5′-AAATTTCCCCATACTAAAACTACGACG-3′<br>NKD1-U- Reverse: 5′-TAAAATTTCCCCATACTAAAACTACAACA-3′<br>NKD2-M- Forward: 5′- GTTGTCGTCGTTGTTTTCGCGTTTTGC-3′<br>NKD2-U- Forward: 5′- GTTGTTGTTGTTGTTGTTTTTGTGTTTTGT-3′<br>NKD2-M- Reverse: 5′- AAATAAAAAAAAATAACTCCACGCCGCG -3′<br>NKD2-U- Reverse: 5′- AAAAAATAAAAAAAAATAACTCCACACCACA -3′
**bisulfite sequencing primers**
NKD2- Forward: 5′-GTTGGTGGGGTTTTAGGTTGG-3′<br>NKD2- Reverse: 5′-AACTAAATTCTAAAACCRAAACC-3′
